# High-pressure torsion of biodegradable Mg−Zn−Mn alloy and investigate mechanical and corrosion behaviour

**DOI:** 10.1038/s41598-025-20031-8

**Published:** 2025-10-16

**Authors:** Prakash Kumar, Gajanan Anne, S. Ramesh, S. Aditya Kudva, M. R. Ramesh, Mrityunjay Doddamani, Ashwini Prabhu, Sandeep Sahu

**Affiliations:** 1 UnivLabs Technologies Pvt. Limited , Gurugram, Haryana 122003 India; 2 Department of Mechanical and Industrial Engineering, Manipal Institute of Technology, Manipal Academy of Higher Education , Manipal, Karnataka 567104 India; 3 School of Computer Science and Engineering , RV University , Bengaluru, 560059 India; 4 Department of Mechanical Engineering , Shri Madhwa Vadiraja Institute of Technology and Management , Udupi, 574115 India; 5 Department of Mechanical Engineering , National Institute of Technology Karnataka , Surathkal, Mangaluru, 575025 India; 6 Mechanical Engineering , Indian Institute of Technology, Jodhpur District, Jodhpur, Rajasthan 342030 India; 7 Yenepoya Research Centre , Yenepoya (Deemed to Be University) , Deralakatte, Mangalore, India; 8 School of Mechanical and Materials Engineering , Indian Institute of Technology , Mandi, Himachal Pradesh 175075 India; 9 Department of Mechanical Engineering , University of Southampton , Southampton, SO17 1BJ UK

**Keywords:** Mg–Zn–Mn alloy, HPT, Corrosion behaviour, Cytotoxicity, Engineering, Materials science

## Abstract

Considering their biodegradability in physiological environments and similar elastic modulus to natural bone, magnesium alloys have generated a lot of interest as biodegradable implant materials. Their poor corrosion resistance is primarily a result of the inhomogeneous distribution of their second phase, which limits their clinical application. High pressure torsion (HPT) one of the severe plastic deformation techniques which provides an opportunity to process materials with low formability such as magnesium at room temperature. The present study HPT is conducted for Mg-Zn-Mn alloy up to ten revolutions at room temperature. Optical, scanning, and transmission electron microscopes were used to examine the microstructures of base material (BM) and ten revolution HPT samples. Significant microhardness improvement was observed in HPT N10 samples (222 Hv) as compared to BM samples (68 Hv). It was determined that the improvement in microhardness was primarily due to dislocation strengthening, fine grain strengthening, and second phase strengthening. Potentiodynamic polarisation and electrochemical impedance spectroscopy (EIS) were used in a simulated body fluid (SBF) solution to assess the corrosion behaviour. When compared to the BM sample (0.0243 mm/y), the corrosion resistance of the HPT N10 sample (0.0012 mm/y) increased significantly. This was mostly due to the smaller grain size and uniform dispersion of the secondary phases, which result in a uniform corrosion. Further, obtained data from the cytotoxicity assay carried out using the MTT method indicated the compatibility of the Mg-Zn-Mn alloy on MG-63 osteoblast-like cells, further substantiating its safety on the bone cells.

## Introduction

Much work has been done over the past 20 years to develop new biodegradable magnesium alloys that can be used in bone-fixing implants^1–3^. The area of medicine has made rapid progress in the creation of biological implants that are non-toxic, biodegradable, and compatible with the complex human body thanks to recent advancements in material science that have made it possible to employ promising materials^4^. Out of all the alloys that have been studied, magnesium alloys have significant benefits over other resorbable materials and permanent biomaterials^5,6^. The density and tensile strength of magnesium alloys are comparable to that of human bone, making them practically perfect candidate materials for fracture treatments^7^. More significantly, magnesium is essential to human metabolism and is the fourth most prevalent cation in the body. Therefore, the recommended daily intake of magnesium for an adult is between 240 and 420 mg, with any excess cations being removed harmlessly in the urine^8,9^. Even with a biodegradable implant, the primary obstacle to using magnesium and magnesium alloys as biomedical materials is their extremely rapid rate of deterioration in physiological and aquatic conditions. Osteolysis and the balloon effect, which delay bone tissue repair, have resulted in several issues, including decreasing the mechanical integrity of the implant before bone healing ends^10^. Many techniques, such as alloying^11,12^, heat treatment^7^, coating^13,14^, and severe plastic deformation (SPD)^15–19^, have been used to improve the properties of magnesium alloys.

HPT is regarded as one of the SPD procedures that may significantly improve the mechanical characteristics and corrosion behaviour of the materials as results of refining the structure^20^. An investigation of the effect of solution pretreatment on the homogeneity and corrosion resistance of biomedical Mg-Zn-Ca alloys treated by high pressure torsion was conducted by Zhang et al.^21^. Rojas et al. (2025)^22^ studied the microstructure and mechanical behavior of a bioabsorbable Mg–1Ca alloy processed by high-pressure torsion. After 10 revolutions, the alloy showed notable improvements, with microhardness rising from 58 to ~ 62 HV, UTS increasing from 186 to 199 MPa, and elongation improving from 11% to 30%. These enhancements were mainly attributed to grain refinement and the intense plastic deformation introduced by HPT. As the number of revolutions increases, the corrosion resistance of the HPT-treated Mg-Zn-Ca alloy increases gradually, according to the authors. The Mg-9%Al alloy was shown to be superplastic at a temperature of 473 K, and average grain sizes found to be 0.51 μm after five revolutions HPT treatment, according to Kai et al.^23^. K. Zhao et al.^24^ state that it is critical to investigate the effects of HPT since the distribution of the second phase significantly affects magnesium alloys corrosion behaviour. According to Parfenov et al.^25^, HPT decreased the Mg-1Ca (X1) alloy’s grain size from 42 μm to around 100 nm, and grain refinement improved the alloy’s resistance to corrosion. The annealing process after deformation resulted in the precipitation of nano-sized Mg_2_Ca particles and the dissolution of the large Mg_2_Ca phase, which further decreased the corrosion rate. Gao et al. 2011^26^ The corrosion behavior of the as-cast, extruded, and HPT-treated Mg–Zn–Ca alloys was examined through potentiodynamic polarization. The HPT-treated alloy exhibited a remarkable decrease in corrosion current density, dropping from 5.3 × 10⁻⁴ A/cm² in the as-cast state to 3.3 × 10⁻⁶ A/cm², while its corrosion potential shifted slightly toward more negative values. To date, literature has been more focused on ultrafine-grained Mg alloys prepared by HPT and more focused on effect factors on texture evolution, deformation behavior, and mechanical properties^27^. The understanding of the distribution of plastic deformation and evolution of structure during HPT processing is of key importance to the future production of components of magnesium alloys with superior strength, and improved corrosion resistance. Ren et al. (2022)^28^ examined the microstructural evolution and hardness of an Mg-Gd-Y-Zr alloy subjected to high-pressure torsion. After five HPT revolutions, the alloy developed a nanocrystalline structure, and the hardness at the disk edge reached ~ 120 Hv, which is higher than that obtained through conventional plastic deformation techniques. However, investigations of the synergistic effect of HPT on microstructure modification, mechanical properties, and degradation behavior of Mg-Zn-Mn alloy have rarely been found so far^29^. The present paper aims to reveal the effect of HPT on the microstructure, mechanical properties, biodegradation behavior, and cytocompatibility of Mg-Zn-Mn alloy.

## Materials and methods

### Material preparation

Mg-Zn-Mn alloy was cast using permanent mold casting done in Vision casting Hyderabad India. An electrical resistance furnace was used to melt pure magnesium (99.9 weight%) ingots as the base metal and pure zinc and manganese (99.9 weight%) granules as the alloying elements. The temperature range for the melting process was 750 to 800 °C, with an inert environment of 99% CO_2_^30^. To reduce the amount of impurities in the molten alloy, mechanical agitation was used during the melting process. The blocks were heated to 300 °C for 24 h to remove the dendritic microstructure of the as-cast alloy and create a homogenous microstructure. Using a wire-cut electric discharge machine, blocks measuring 100 × 50 × 10 mm were machined from the as-cast alloy. These blocks are regarded as base materials (BM) for further study. Chemical spectroscopy was used to determine the composition of the Mg-Zn-Mn alloy, which contained 4.23% Zn,1.18% Mn, and the remaining Mg.

### HPT processing

The sample thickness was further lowered by mechanical grinding with SiC abrasive paper, from 1 mm to 0.88 mm, prior to the HPT procedure. The HPT setup is made up of two anvils with a circular cavity of 10 mm in diameter and 0.25 mm in depth^31^. samples were inserted into the lower anvil chamber, and for one minute at room temperature, a hydraulic pressure of six GPa was applied, compressing the disc between the upper and lower anvils. It was decided to balance processing conditions with maintaining the original properties of the materials, which led to the choice of performing HPT at room temperature. The lower anvil revolved anticlockwise for varying amounts of revolution 1/4, 1/2, 1, 5, and 10 at room temperature while the top anvil stayed locked in place during HPT processing. After ten revolutions, the sample’s thickness was around 0.7 mm. Equation (1) gives the equivalent strain ($\:{}_{eq}$), as established by the von Mises yield criteria^32^.

1 $$\:{}_{eq}=\frac{2\pi\:Nr}{1\sqrt{3}}$$

Whereas, N- number of revolutions, r- distance from the rotation axis at the disc centre. Table 1 displays equivalent strain measurements that were made without slippage during HPT, collected at a radial distance *r* = 4.5 mm from the centre.

**Table 1:** Mg-Zn-Mn condition prior to and after HPT processing abbreviation and the associated von mises strain.

Sample conditions	Sample Abbreviations	Von Mises strain (ε)
Homogenized Mg-Zn-Mn	BM	0
1/4 revolution HPT processed Mg-Zn-Mn	HPT N¼	4.1
1/2 revolution HPT processed Mg-Zn-Mn	HPT N½	8.2
1 revolution HPT processed Mg-Zn-Mn	HPT N1	16.5
5 revolution processed Mg-Zn-Mn	HPT N5	82.4
10 revolution HPT processed Mg-Zn-Mn	HPT N10	164.9

### Microstructural analysis

Microstructural characterisation of the HPT processed material was performed in the centre portion, transverse cross section of the samples was prepared using conventional metallographic process, that involves the mounting of the samples followed by manual polishing. The sample was polished with 0.25 μm diamond paste, etched with picral reagent, then dried with ethanol and compressed air to produce a mirror-like surface free of scratches. Energy-dispersive spectroscopy (EDS) was used to assess the localized chemical composition while scanning electron microscopy (SEM) (ZEISS-EVO MA18 with Oxford EDS) and the optical microscope (OM-Olympus) was used for microstructure investigation. The various phases of the BM and HPT samples were determined by X-ray diffraction (XRD) (Rigaku Miniflex 600 (5th gen)) using Cu Ka radiation for the Bragg angle, 30° ≤ 2θ ≤ 90° using step size of 2°/min.

### Vickers microhardness

Vickers microhardness (Digital Micro – MMT X – Chennai Metco) was measured over the diameter of the HPT disc for 15 s with a load of 100 gf. Each point reflects the average of four measurements spaced 500 μm apart.

### Corrosion studies

Potentiodynamic polarisation and EIS were conducted as per ASTM G59 standard using an AC Gill, UK electrochemical workstation. This test makes use of three electrodes: a platinum electrode, a saturated calomel electrode, and a sample. Open circuit polarization is carried out for 10 min for stabilization and followed by EIS and polarization tests. The voltage that is being applied is between − 250 and + 250 mV and scan rate was 5mV/min. EIS was carried out between 100 kHz and 0.1 Hz using an amplitude of 10 mV using simulated body fluid (SBF) solution.

2 $$\text{CR} (\text{mm/y}) =\:3.27\:X{10}^{-3\:}\frac{Icorr\:X\:A}{\rho\:}$$

In this case, CR is the corrosion rate, Icorr is the corrosion current density, ρ is the density, and A is the molar mass. Based on Tafel extrapolation of the anodic and cathodic curves corrosion potentials (Ecorr) and corrosion current densities (Icorr) were determined for the samples.

### Cytocompatibility test

MG-63 osteoblast-like cells were used to assess the test implants’ effects using the Methyl Thiazolyl Tetrazolium (MTT) assay. Dulbecco’s modified Eagle’s Medium (DMEM) was supplemented with 10% fetal bovine serum (FBS) and 1% antibiotic-antimycotic solution for cultivation of the cells. The cells were incubated at 37 °C in a CO_2_ incubator in a humid atmosphere. Upon achieving a confluency level of 70%, they were sub-cultured using trypsinisation and utilized for the cytocompatibility testing, after three consecutive passages. MTT assay was performed using MG-63 cells as per the modified protocol outlined by Mosmann, 1983^33^. A density of 5000 cells per well was used to seed them onto 96-well microtiter plates, which were subsequently incubated at 37 °C and 5% CO_2_. Once attached, the cells were exposed to the bare Mg-Zn-Mn alloy and HPT samples for 24 h. The spent media was taken out of the wells after treatment, and 100 µL of MTT reagent (1 mg/mL) was added. The wells were then incubated for a further four hours. A multimode microplate reader (FluoSTAR Omega, BMG Labtech) was used to measure absorbance at 570 nm of the formazan crystals dissolved in DMSO. Untreated cells served as control, against which the cytocompatibility of the test implants was calculated. The results were expressed as Relative percentage of cell proliferation in terms of mean ± SD (*n* = 3). The statistical significance between the test group and control was examined using the Paired T-test. When *P* < 0.05 was reached, the results were deemed significant.

## Results and discussion

### Microstructural analysis

**Fig. 1:**
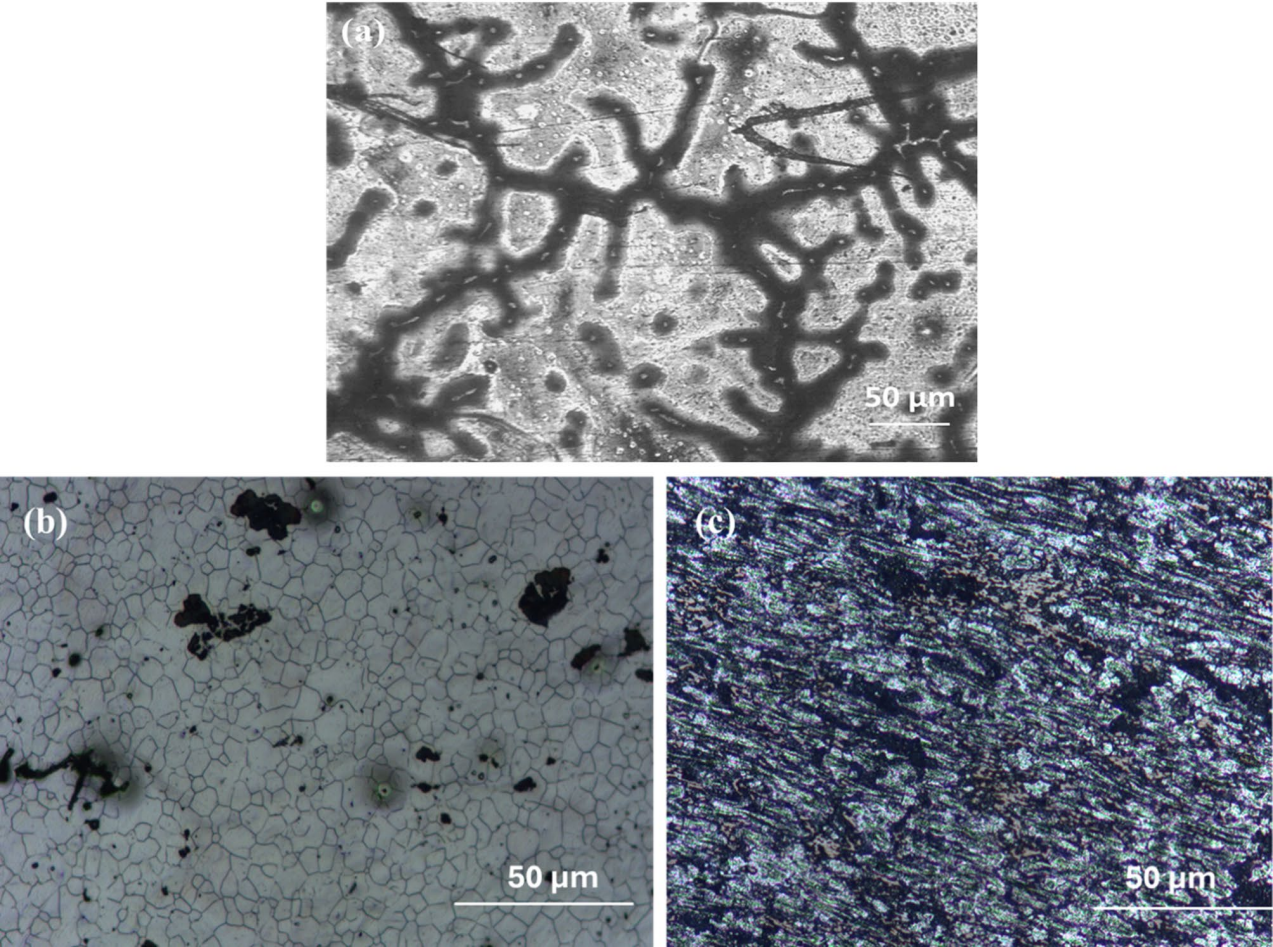
Optical image of the (a) BM (b) HPT N1 (c) HPT N10 samples.

The microstructures of the BM sample are presented in Fig. 1 (a), observed dendritic structure consists of course grains. A Linear intersect method was used to determine the average grain size, which was 246± 7 μm. BM sample are primarily composed of the α-Mg phase, with Zn and Mn (β phases) mainly dispersed along the grain boundaries.

**Fig. 2:**
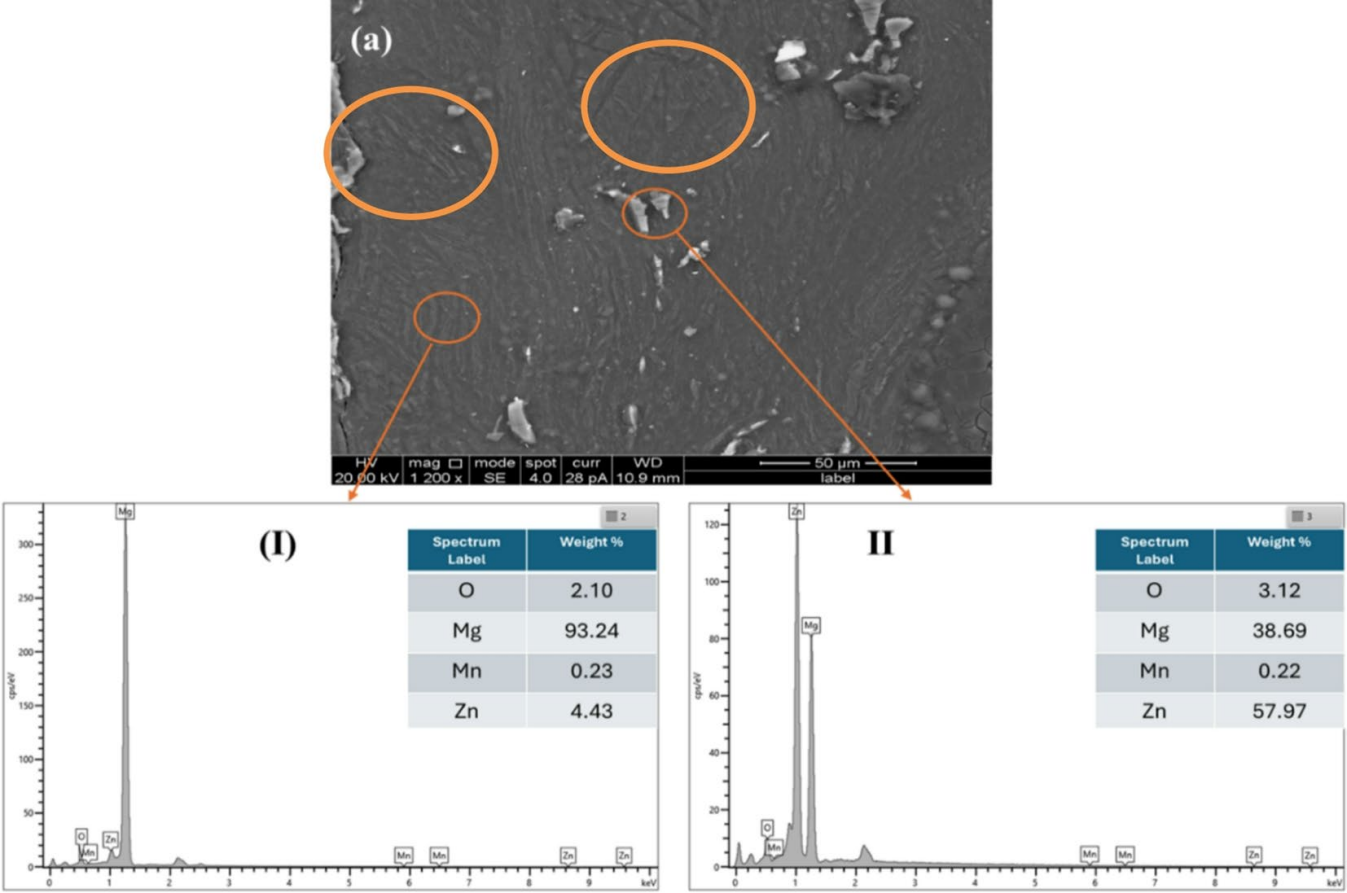
SEM images of (a) HPT N10 sample (I) and (II) corresponding to EDS analyses.

As shown in Fig. 1(b), the HPT N1 sample (*r* = 4.5 mm from the canter) demonstrates a fine grain structure reduced from 246 to 21 ± 3 μm. It is evident that the discs undergo substantial compression and shear deformation throughout the specimen diameter, Fig. 1(c) shows the HPT N10 sample (*r* = 4.5 mm from the center), grain size found to be 8 ± 0.5 μm. Grain boundaries collapse and the material becomes severely distorted as the strain increases. The HPT N10 samples underwent SEM and TEM analysis in order to better understand the microstructural changes. The SEM images of the HPT N10 samples and the accompanying EDS analysis are displayed in Fig. 2. Figure 2. (a) depicts twins and subgrains that were observed in some portions of the microstructure, which are highlighted in orange circles. Grain size and crystallite size were found to decrease with increasing deformation, as the imposed severe plastic deformation intensified with a higher number of HPT revolutions. The alloy processed by HPT exhibited significant grain refinement due to the high strain introduced during the process.

**Fig. 3:**
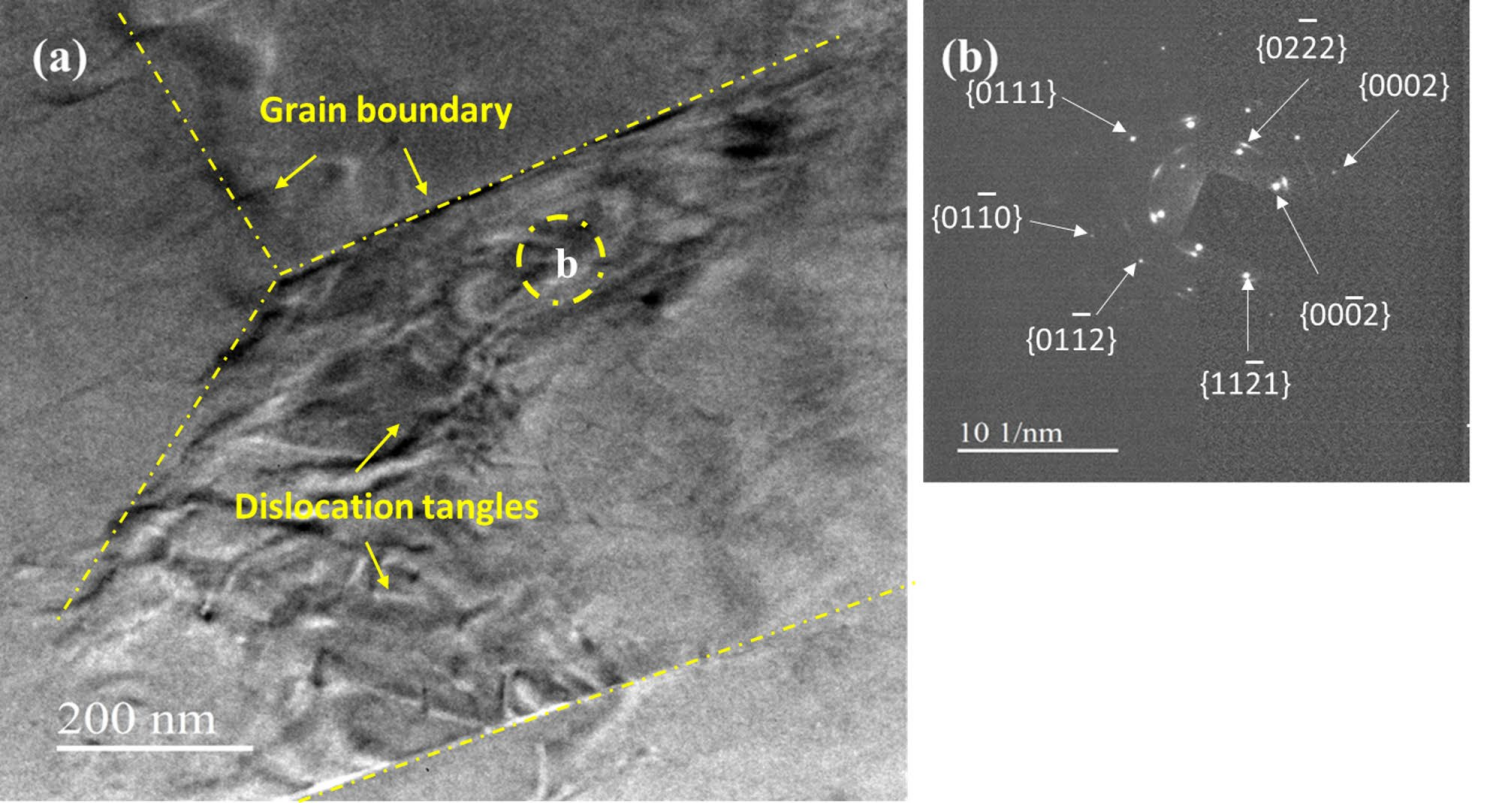
TEM images of the (a) HPT N10 sample and (b) SAED pattern.

Figure 3 (a) depicts the HPT N10 treated alloy was subjected to TEM examinations, ultra fine grains were observed. The corresponding SAED pattern (Fig. 3(b)) indicates that some of the low-angle subgrains have evolved into high-angle grain boundaries, leading to the development of nanocrystalline grains, as confirmed by the sequence of distinct concentric rings corresponding to the α-Mg matrix. Distinguishing distinct nanoscale precipitates is challenging due to the high density of sub-grains and dislocation structures^34^. Moreover, research has shown that the combined addition of Zn and Mn reduces stacking fault energy and increases dislocation density^35^. During HPT processing, an increase in stacking fault width inhibits dislocation cross-slip, facilitating dislocation interactions and enhancing strain hardening^36,37^.

### X-ray diffraction analysis

**Fig. 4:**
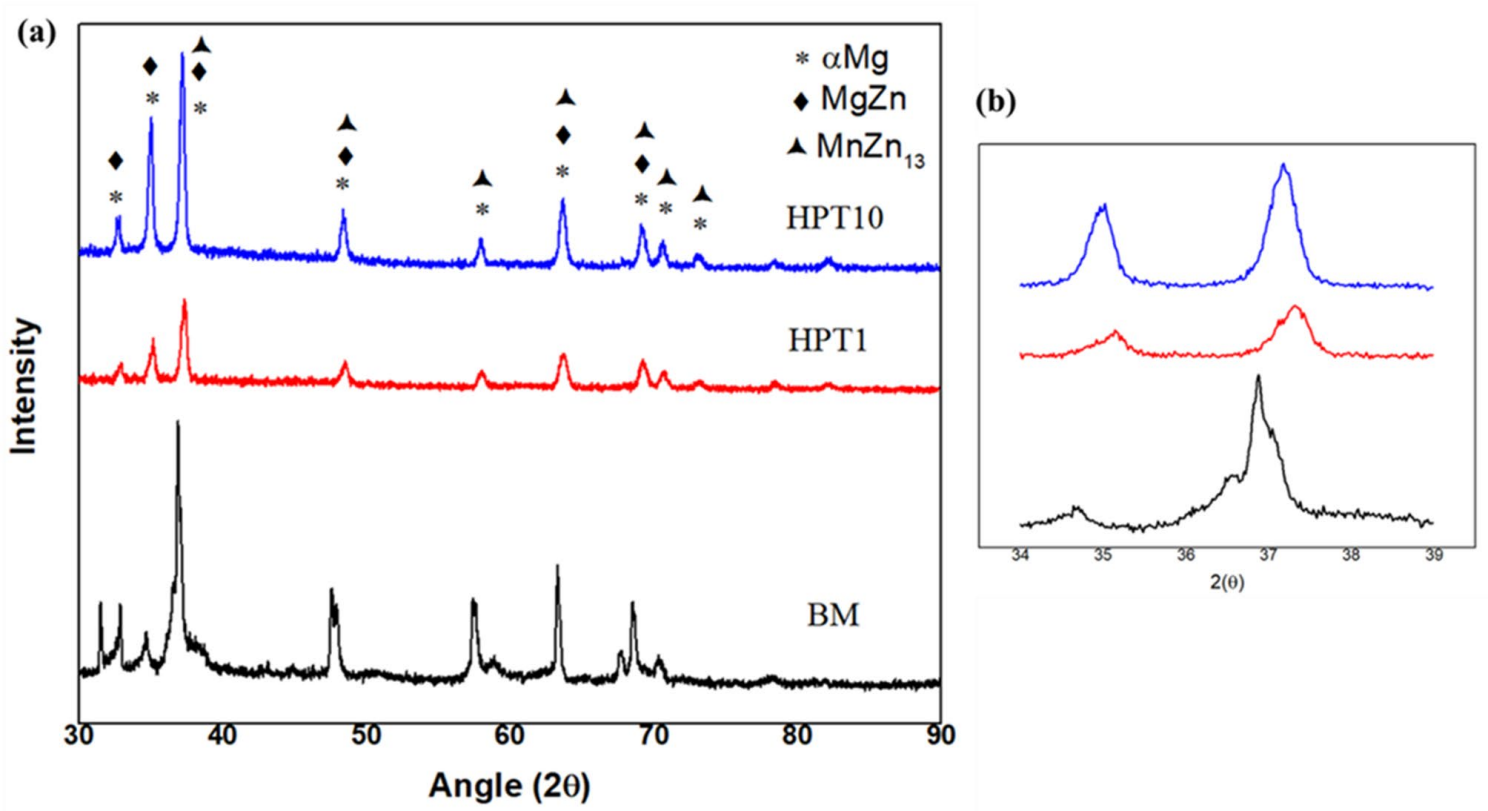
(a) X-ray diffraction patterns of BM, HPT N1 and HPT N10 samples (b) magnified XRD patterns.

X-ray diffraction analysis (Rigaku Miniflex 600 (5th gen)) was carried out using the target of Cu kα with the angle range of 30° ≤ 2θ ≤ 90 ° in steps of 2°/minute. Figure 4 (a) shows the XRD spectra of Mg-Zn-Mn alloy before (BM) and after HPT processing (HPT N1 and HPT N10). The peaks of αMg MgZn and MnZn_13_ phases were identified during XRD analysis. Magnified XRD patterns showing peak shifting and broadening are depicted in Fig. 4 (b). Peak broadening could be attributed to the structural refinement or incorporation of lattice strains. This result confirms that high-pressure torsion is one of the severe plastic modification techniques which induces the refinement of grains and lattice strain in bulk materials^38,39^. With an increasing number of HPT revolutions, the XRD peaks broaden without the appearance of new peaks. This peak broadening is attributed to grain refinement and the build-up of internal stresses (lattice strain) induced by severe plastic deformation^40,41^.

### Micro hardness

**Fig. 5:**
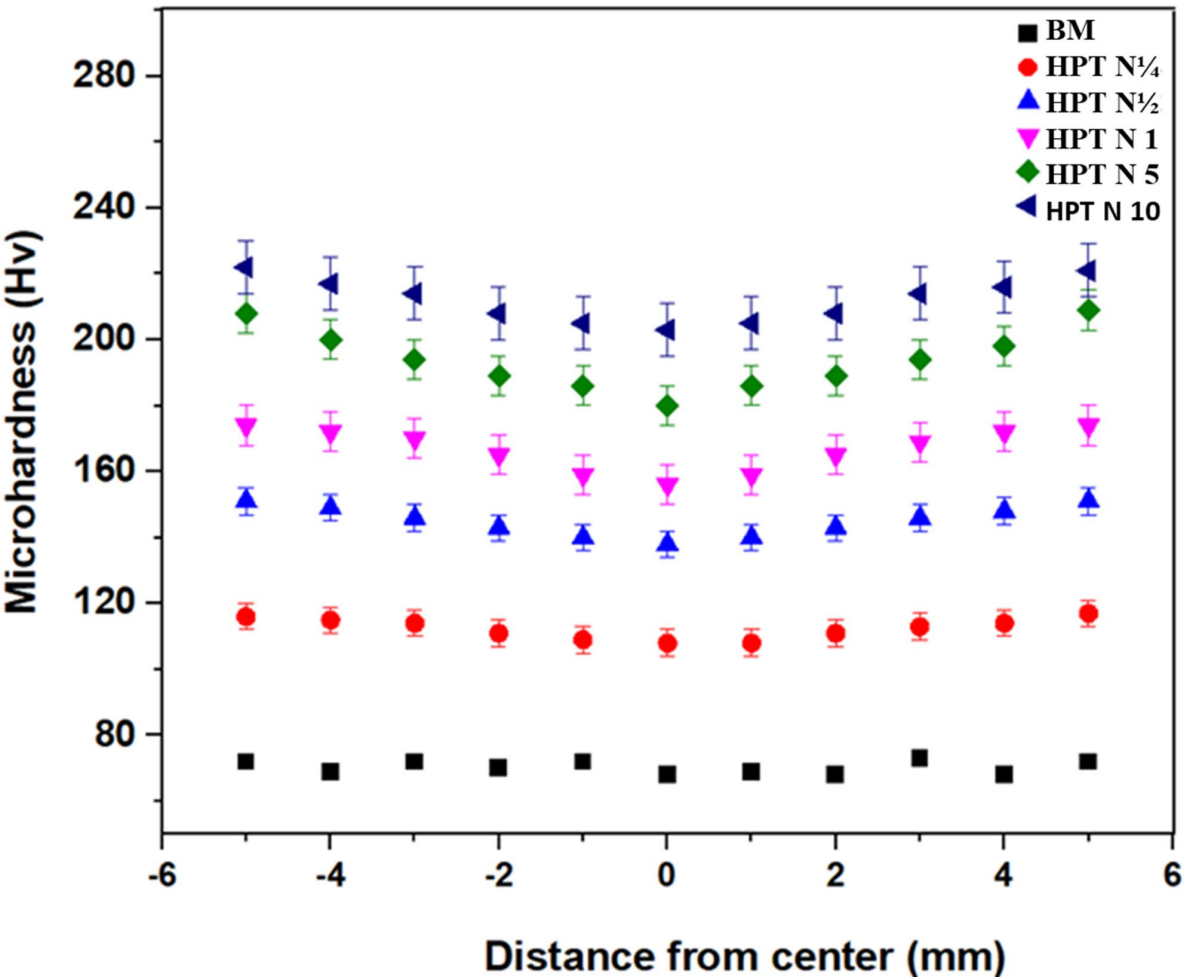
demonstrates the microhardness distribution along the diameter of the BM sample and the samples that have undergone HPT processing.

Vickers microhardness is shown in Fig. 5 at various points on the discs, with the disc centre having the lowest hardness in all HPT samples. The figure in the error bars show how the microhardness levels vary at different locations. A significant increase in the hardness of the alloy treated by HPT with more revolutions than the BM was seen, as shown in Figure. After HPT processing, the alloy’s hardness increased more quickly near the edges than at the centres of the disc-shaped samples. Prior to HPT processing, the BM sample had an average hardness of 68 Hv. The strain distribution on the disc, which rises from the centre to the edge, is consistent with the symmetrical distribution of hardness along the diameter. The average achieved hardness value after 10 HPT revolutions was around 222 Hv. Later, saturation of hardness was seen for larger numbers of HPT revolutions at both the centre and peripheral areas. A tendency of increased microhardness with varying numbers of revolutions is observed in samples treated by HPT, extending from the disc’s centre to its periphery. During HPT deformation, shear strain accumulates across the diameter, which is essentially consistent across a wide range of alloys processed with HPT^42^. The average microhardness progressively increases with the number of HPT revolutions, mostly due to strengthened grain boundaries, dislocations, and solid solutions strengthening. When stress is applied to grain boundaries (GBs), a lot of dislocations may become activated and migrate along slip planes, which will result in a lot of stress concentrated at GBs. As a result, adjacent grains near grain boundaries may experience dislocations. The moving dislocations cut the cluster bonds located at grain boundaries, increasing hardness.

### Wettability measurement

**Fig. 6:**
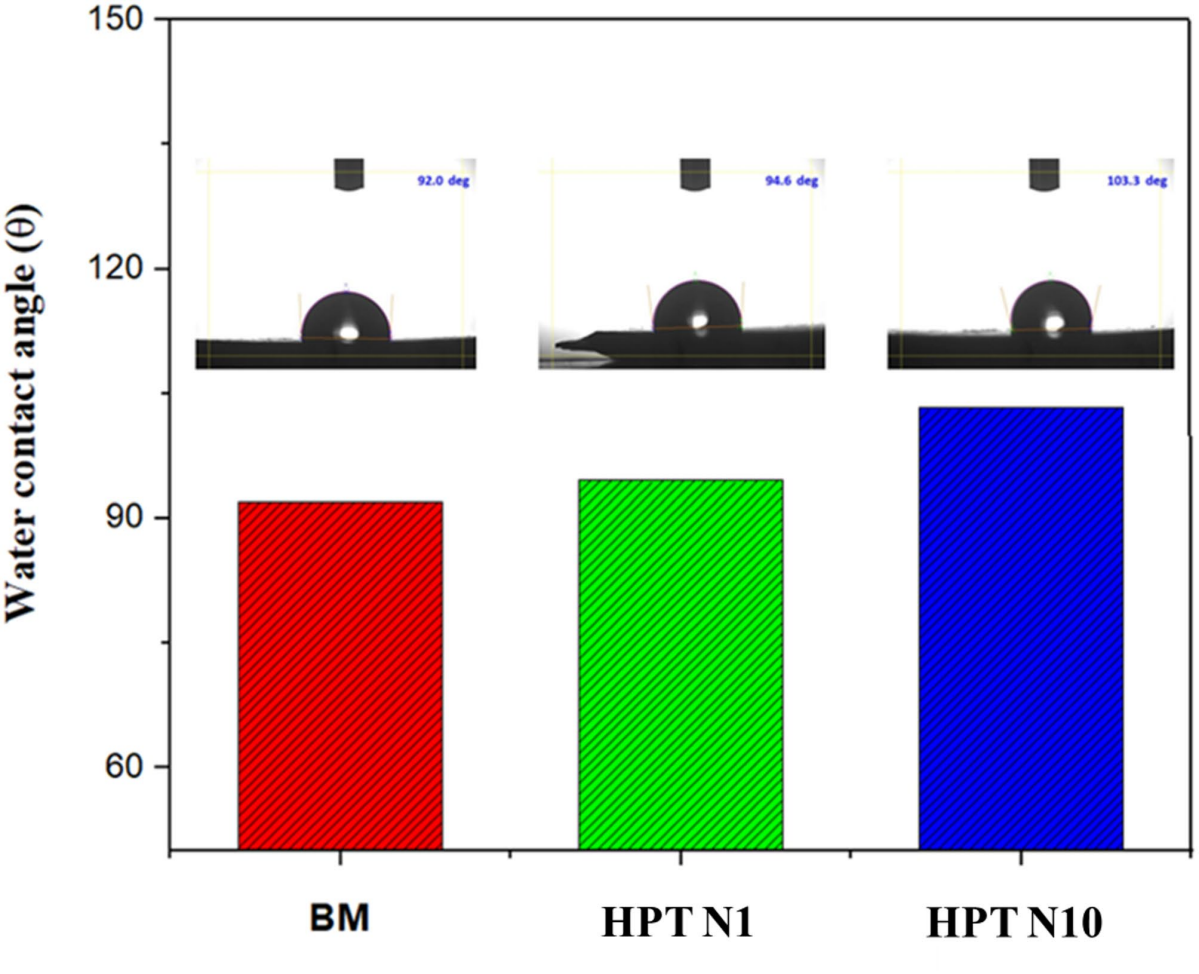
Contact angle of the BM and HPT processed samples.

Schematic representations of the contact angles of distilled water on BM samples and HPT treated samples are shown in Fig. 6. For the BM, HPT N1, and HPT N10 samples, the contact angles are determined to be 92°, 94.6° 103.3° respectively. It is evident from the graph that the contact angle increases with the number of revolutions. It can be observed that the BM sample has less hydrophobicity than the HPT N1 samples, whereas the HPT N10 samples have more. Pressure torsion refines the microstructure of Mg alloys by producing ultra-fine grains. This grain refinement can lead to a higher density of grain boundaries, which can affect surface energy and, consequently, hydrophobicity. After SPD processing, it is common to observe changes in the contact angle due to the altered microstructure and surface characteristics. Improved hydrophobicity in Mg alloys can be beneficial for applications in automotive, aerospace, and biomedical fields, where corrosion resistance is critical. Kim et al.^27^ revealed a trend that may be attributed to the surface energy differential, which is influenced by the grain size distribution and surface microstructure.

### Corrosion behaviour

**Fig. 7:**
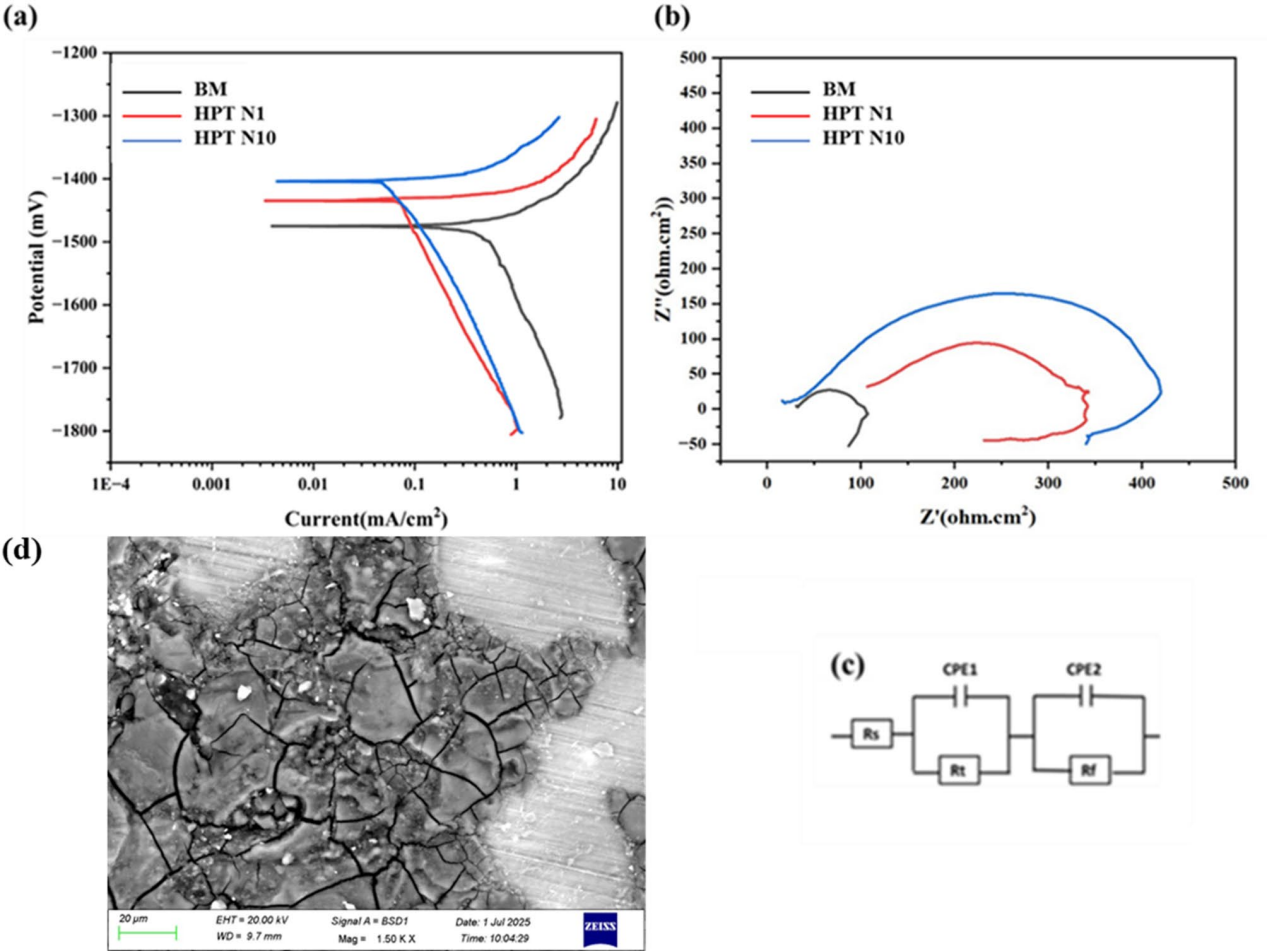
Electrochemical corrosion data (a) Potentiodynamic polarisation curves (b) Nyquist plots (c) equivalent circuit (d) surface morphology after electrochemical corrosion test.

The observed potentiodynamic polarisation curves of the BM and HPT processed sample in SBF solution at 37 °C are displayed in Fig. 7(a). Table 2 compiles the Ecorr and icorr values derived from Tafel curves. The *E*_*corr*_ is − 1473 mV in the BM condition, and it shifts to a noble value of − 1434 mV and − 1401 mV for the HPT N1 and HPT N10 samples respectively. The variation in corrosion current density can reflect the change of corrosion rate which follows the sample trend as BM (0.5461 µA/cm^2^) > HPT N1 (0.2370 µA/cm^2^) > HPT N10 (0.0241 µA/cm^2^). This suggests that the HPT samples showed a relatively low corrosion tendency when compared to the BM sample, increasing the deformation (HPT revolutions) clearly demonstrated a shift in the cathodic currents to the lower current densities. Furthermore, the cathodic branches of the HPT samples shift to lower current density direction and potential moving towards more positive indicating better corrosion resistance. Corrosion rate of the different sample is calculated according to Eq. 1 and found to be 0.0243 mm/y, 0.0105 mm/y and 0.0012 mm/y for the BM, HPT N1 and HPT N10 sample. Corrosion rate of the HPT N10 sample exhibited better corrosion resistance compared to HPT N1 and BM samples mainly due to the combined effect of fine grain structure, uniform distribution of secondary elements and also compressive residual stress acting on the materials during HPT process^43–46^.

Gao et al. 2011^47^ reported that HPT treatment leads to the uniform distribution of secondary phases, transforming them into nano-sized particles distributed within the grain interiors rather than along grain boundaries. This uniform distribution helps in achieving homogeneous corrosion. Sarraf et al. 2019^48^ investigated electrochemical behavior of a magnesium ZK60 alloy processed by high-pressure torsion. Reported that immersion tests and electrochemical impedance spectroscopy confirm that HPT-treated Mg samples have better corrosion resistance compared to their as-cast counterparts. The improved corrosion resistance of HPT Mg-based samples is attributed to the combined effects of fine grain structure, uniform distribution of secondary elements, and compressive residual stress collectively enhance the material’s ability to form a stable and protective passive film, reduce localized corrosion, and improve overall mechanical properties, thereby contributing to better corrosion resistance. Figure 7(b) presents the Nyquist plots of the specimens in the BM and HPT conditions. The HPT N10 sample displays the largest capacitive arc followed by HPT N1, and BM depicts the smallest capacitive arc. The diameter of the semicircle is associated with the charge-transfer resistance, higher the semicircle maximum will be the corrosion resistance and an inductive arc suggesting adsorption of the ionic species or pitting formation during the test. Number of HPT revolution has significant effect on the curves for these alloys which is proportional to the polarization resistance. In this circuit (Fig. 7c), *Rsl* represents the solution resistance, *Cdl* represent the double layer capacitance, and *Rct* denotes charge transfer resistance, respectively. Table 2 reveals that HPT N1 displays the highest Rct compare to BM sample but HPT 10 indicating highest value and exhibits superior corrosion resistance. The second phase particles tend to scatter evenly while the grain size of HPT N10 material is very fine, and the density of GBs grows concurrently. Corrosion mechanism is controlled not only by a charge-transfer process but also by the diffusion of charged species through the corrosion products. As seen in Fig. 7(d), corrosion happens in the areas close to the second phases, GBs, dislocation tangle zones, and sub grains when HPT material comes into contact with SBF. HPT material demonstrated not shown any corrosion pits, and a homogeneous and compact layer of corrosion product developed (MgO/Mg(OH)_2_). HPT material depicted typical uniform corrosion degradation characteristics^49^. Corrosion resistance increases after deformation due to decreased grain size and homogenization of precipitate distributions during HPT^50–53^.

**Table 2:** Polarisation and impedance spectroscopy curve results.

Tafel data				Nyquist data		
Sample	E_*corr*_ (mV)	i_*corr*_ (µA/cm^2^)	Corrosion Rate mm/y	Rsl (Ohms. Cm^2^)	Rct (Ohms Cm^2^)	Cdl (F/cm^2^)
BM	−1473	0.5461	0.0243	9.274E + 01	2.562E + 02	1.257E-05
HPT N1	−1434	0.2370	0.0105	5.696E + 01	2.900E + 02	4.238E-05
HPT N10	−1401	0.0241	0.0012	1.393E + 01	4.937E + 02	4.393E-05

### Cytocompatibility

Results of cytocompatibility testing showed that the developed material is found to be compatible on MG-63 osteoblast-like cells. In comparison to the BM sample, HPT N10 samples promoted the proliferation of osteoblast cells, indicating the possible potentiality of the implant material in bone tissue regeneration. Bare uncoated composites exerted cytotoxicity on the tested cells, probably due to the excessive concentration of Zn ions, which contribute to the increase in the intracellular and extracellular osmotic pressure in the cells and increase the cellular pH^54^. The results of cytocomaptibility testing of the candidate composites are represented in Figure- 8. HPT samples induced cell proliferative behavior, with a physiological pH compatibility and lowered ionic concentrations. In addition to having appropriate in vitro degradation characteristics and good mechanical qualities that suit for application on human bone defects, a biodegradable bone-implant metal must also have good cytocompatibility on the osteoblast cells^55^. By boosting the early mineralization, zinc ions can support osteogenic differentiation and promote bone formation and regeneration^56^. Magnesium ions are known to possess a significant osteogenic potential by enhancing the proliferation of osteoblastic cells^57^. There are reports on the reversal of Zn^2+^ toxicity by the addition of Mg2 + during the preparation of composites for biomedical applications. As per the ISO 10,993 standards, materials displaying more than 70% cell viability are known to exert a desired cytocompatibility. In our study, the tested HPT composites showed a cell proliferation of > 120% relative to the cell control, which indicates an excellent cytocompatibility of the material on MG-63 osteoblast-like cells.

**Fig. 8:**
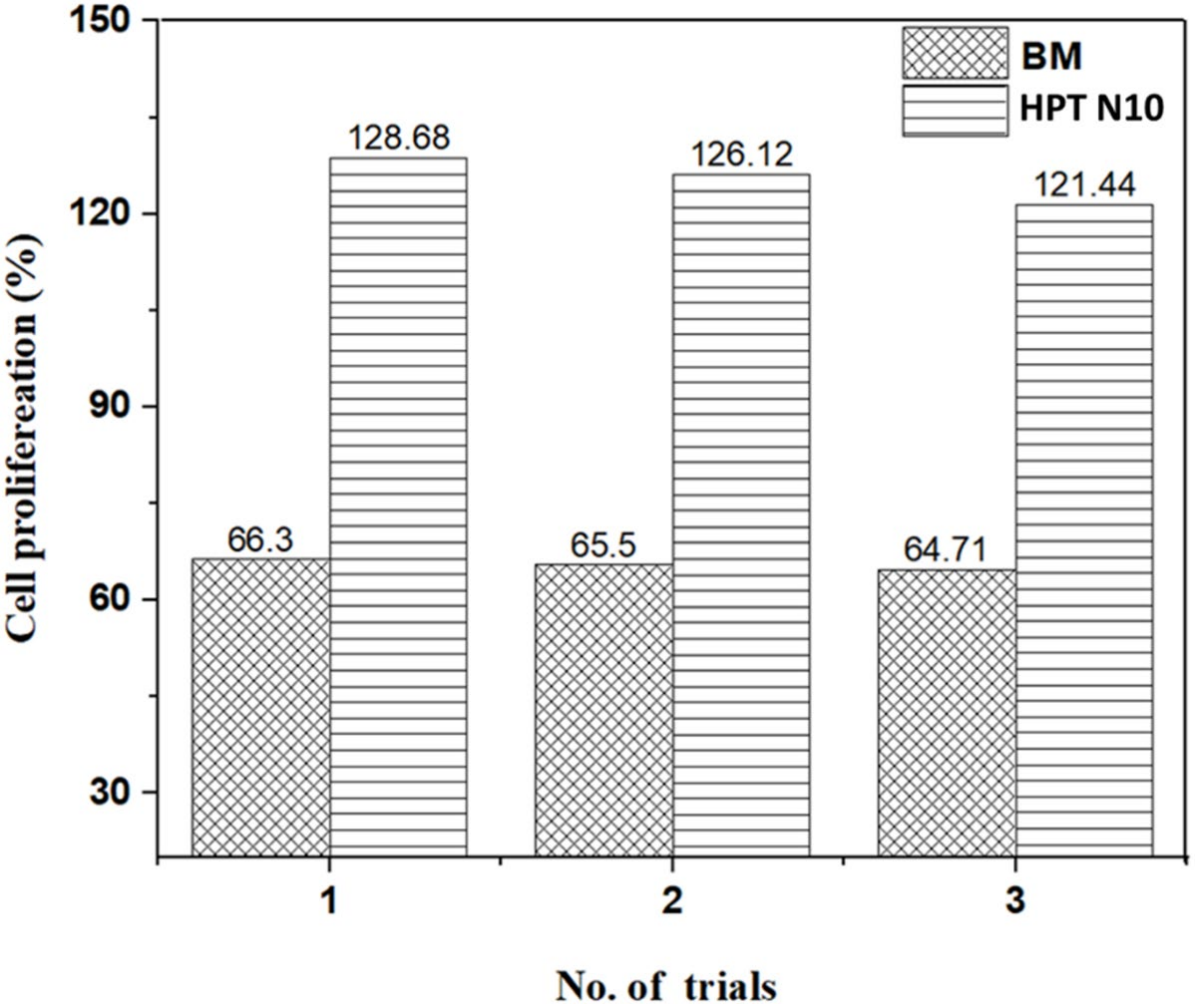
BM and HPT processed samples exerted cytotoxicity on the MG-63 cells tested.

## Conclusions

As the number of HPT revolutions increases, the grain size decreases, and the microstructural homogeneity improves. The average grain size of the BM sample was approximately 246 ± 4 μm, decreasing to 8 ± 0.5 μm after 10 revolutions of HPT process.

The hardness gradually increases with the increase of equivalentstrain and average hardness of the HPT N10 sample found to be 222 Hv which is 3.26 times higher as compared to BM samples.

XRD patterns indicate the presence of αMg MgZn and MnZn_13_ phase in both BM, HPT N1 and HPT N10 samples and as the number of HPT revolutions increases peak broadening and shifting are observed.

HPT processing greatly enhances Mg alloy corrosion resistance, lowering the corrosion rate from 0.0243 mm/y (BM) to 0.0012 mm/y (HPT N10) and shifting Ecorr from − 1473 mV to − 1401 mV. This is mainly attributed to grain refinement, uniform secondary phase distribution, and compressive residual stresses, promoting a stable protective layer and uniform corrosion.

Mg-Zn-Mn alloy fabricated using the HPT method was found to possess higher cytocompatibility and cyto-proliferative efficacy on the tested MG-63 osteoblast-like cells, further confirming its promising effects on bone tissue regeneration.

## Data Availability

The datasets used and/or analysed during the current study available from thecorresponding author on reasonable request.
